# Screen-Printed Graphite Electrode Modified with Graphene-Co_3_O_4_ Nanocomposite: Voltammetric Assay of Morphine in the Presence of Diclofenac in Pharmaceutical and Biological Samples

**DOI:** 10.3390/nano12193454

**Published:** 2022-10-03

**Authors:** Hadi Beitollahi, Fraiba Garkani Nejad, Somayeh Tajik, Antonio Di Bartolomeo

**Affiliations:** 1 Environment Department, Institute of Science and High Technology and Environmental Sciences, Graduate University of Advanced Technology, Kerman P.O. Box 76318-85356, Iran; 2 Department of Chemistry, Faculty of Science, Shahid Bahonar University of Kerman, Kerman P.O. Box 76175-133, Iran; 3 Research Center of Tropical and Infectious Diseases, Kerman University of Medical Sciences, Kerman P.O. Box 76169-13555, Iran; 4 Department of Physics “E.R. Caianaiello”, University of Salerno, 84084 Fisciano, Salerno, Italy

**Keywords:** electrochemical sensor, morphine, diclofenac, graphene-Co_3_O_4_ nanocomposite, screen-printed graphite electrode

## Abstract

This work focuses on the development of a novel electrochemical sensor for the determination of morphine in the presence of diclofenac. The facile synthesis of graphene-Co_3_O_4_ nanocomposite was performed. The prepared material (graphene-Co_3_O_4_ nanocomposite) was analyzed by diverse microscopic and spectroscopic approaches for its crystallinity, composition, and morphology. Concerning the electrochemical determinations, after drop-casting the as-fabricated graphene-Co_3_O_4_ nanocomposite on the surface of a screen-printed graphite electrode (SPGE), their electrochemical performance was scrutinized towards the morphine detection. It was also found that an SPGE modified by a graphene-Co_3_O_4_ nanocomposite exhibited better electrocatalytic activity for morphine oxidation than unmodified electrode. Under optimal conditions, the differential pulse voltammetry (DPV) was employed to explore the present sensor (graphene-Co_3_O_4_/SPGE), the findings of which revealed a linear dynamic range as broad as 0.02–575.0 µM and a limit of detection (LOD) as narrow as 0.007 μM. The sensitivity was estimated to be 0.4 µM/(µA cm^2^). Furthermore, the graphene-Co_3_O_4_/SPGE sensor demonstrated good analytical efficiency for sensing morphine in the presence of diclofenac in well-spaced anodic peaks. According to the DPV results, this sensor displayed two distinct peaks for the oxidation of morphine and diclofenac with 350 mV potential difference. In addition, the graphene-Co_3_O_4_/SPGE was explored for voltammetric determination of diclofenac and morphine in pharmaceutical and biological specimens of morphine ampoule, diclofenac tablet, and urine, where recovery rates close to 100% were recorded for all of the samples.

## 1. Introduction

Today, pharmaceutical industries and medical sciences have realized the necessity of detecting and quantifying drugs in human biological fluids and pharmaceutical matrices. Morphine, a strong opiate analgesic psychoactive drug, is the prototypical opioid leading to central nervous system (CNS) disorders. Morphine can relieve severe pain in patients, particularly in candidates for surgery [1]. Opium poppy straw is the source for extracting morphine [2]. This substance can act as a precursor in obtaining some opioids, such as codeine, hydromorphone, heroin, dihydromorphine, and nicomorphine [3]. Heroin metabolites can cause the production of this substance, which can subsequently be an indicator of heroin use [4]. In addition, morphine is available as an illegal drug and can be associated with side effects such as hallucinations, slow heart rate, CNS disturbance, constipation, nausea, respiratory problems, muscle stiffness, coma, and dependence [5].

Nonsteroidal anti-inflammatory drugs (NSAIDs) lead to decreased inflammation, pain, and fever throughout the body by inhibiting cyclooxygenase enzymes and reducing the production of prostaglandins [6]. One NSAID is diclofenac, which has potent analgesic, anti-inflammatory, and antipyretic performances. The use of diclofenac is due to effects such as relief of post-traumatic pain, cancer pain, and neuralgia and treatment of osteoarthritis, rheumatoid arthritis, soft tissue disorders, acute gout, and some other inflammatory responses [7,8]. Despite the significant benefits of diclofenac, its inappropriate use can be associated with disadvantages such as extensive liver metabolism, stroke, and hypertension [9]. In addition, the poor degradation of diclofenac in the environment leads to poor water quality and endangers the health of fish [10]. Researchers found that combining morphine and diclofenac in small doses is synergistic in cases of serious inflammatory pains. Actually, using the combination decreases the amount of morphine necessary for sufficient analgesia in comparison to the use of morphine itself [11]. Due to all of these issues, it is vital to detect such analytes in human biological fluids and pharmaceutical matrices.

Accordingly, there have been various analytical ways to detect diclofenac and morphine in diverse matrices, some of which are high-performance liquid chromatography [12,13], chemiluminescence [14,15], capillary electrophoresis [16,17], spectrophotometry [18,19], gas chromatography-mass spectroscopy [20,21], thin-layer chromatography [22], and some electrochemical methods [23,24,25,26]. High selectivity, high sensitivity, and minimal-interference impacts are prominent features of a potent analytical method. Despite the identification of drugs at very low doses by these methods, they require lengthy sample preparation steps, pre-analytical extraction, highly trained technical staff, and expensive instruments. Among these techniques, electrochemical techniques have emerged as important diagnostic tools in recent years, due to their high sensitivity, simplicity, accuracy, and fast detection [27,28,29,30,31].

Special attention in the field of electroanalytical research has recently been drawn to screen-printed electrodes (SPE) in the manufacturing of (bio)sensors [32,33,34,35,36]. Compared to the routine three-electrode system (working, reference, and auxiliary), SPEs have merits such as easier setup, system miniaturization, and portability. Besides the possibility of performing in situ analyses, the ease of mass production, high reproducibility, and low cost make these electrodes very interesting. Chemically modified electrodes (CMEs) are the result of the intentional fixation of a modifying agent on the surface of electrode by various physical and chemical methods [37,38]. Many studies have shown that improvements in the sensitivity and selectivity of electrochemical sensors are achieved due to modifications of the electrode surface [39,40].

Nanomaterials and their applications in various fields have become distinct and active areas of scientific and technological developments over the recent years [41,42,43]. Nanomaterial-supported electrochemical-sensing systems have been considered by many researchers due to their capability for carrying out the electrochemical analysis of diverse analytes resulting from enhanced detection efficacy [44,45,46]. Graphene, a 2D carbon-based nanomaterial, possesses carbon atoms with sp^2^ hybridization connected in a hexagonal lattice architecture. It has admirable electrical conductivity, predominantly because of delocalized π bonds at the top and bottom of its base plane and its huge surface area, and it has recently become a promising candidate for the development of electrochemical sensors [47,48,49,50]. Transition-metal oxides are routine mediators in the construction of electronics and advanced catalysts because of specific traits such as impressive catalytic performance, chemically reactive facets, and great earth abundance [51]. In this regard, cobalt oxide (Co_3_O_4_) has shown commendable physicochemical features such as conductivity, high reversibility, and thermal stabilities [52]. Nanomaterial functionalization remarkably enhances the activity of sensors through the improvement of electrochemical capabilities leading to a greater electron transfer rate compared to a single component. Combining the advantages of the unique features of graphene with Co_3_O_4_ nanoparticles, the graphene/Co_3_O_4_ nanocomposite creates a sensitive and stable base for electroanalysis [53,54].

The present work describes an electrochemical sensor on the basis of SPGE modification with graphene-Co_3_O_4_ nanocomposite for the detection of morphine combined with diclofenac. The resulting modified electrode (graphene-Co_3_O_4_ nanocomposite/SPGE) had a great sensitivity towards the morphine, possessing a narrow LOD and a broad linear range. Furthermore, the co-detection of morphine and diclofenac was performed on the modified electrode surface. The designed graphene-Co_3_O_4_ nanocomposite/SPGE sensor had practical applicability for sensing morphine and diclofenac in real biological and pharmaceutical matrices with acceptable recoveries.

## 2. Materials and Methods

### 2.1. Equipments

An Autolab PGSTAT 320N Potentiostat/Galvanostat Analyzer (Herisau, Switzerland) with GPES (General Purpose Electrochemical System-version 4.9) software was applied for all electrochemical determinations at ambient temperature. The electrochemical sensors were prepared by DRP-110 SPEs (DropSens, Oviedo, Spain) usingsilver pseudo-reference electrode, graphite working electrode, and graphite auxiliary electrode. A Metrohm 713 pH meter with a glass electrode (Metrohm AG, Herisau, Switzerland) was selected to determine and adjust the solutions’ pH. Direct-Q^®^ 8 UV deionized water (Merck Chemicals GmbH, Darmstadt, Germany) was used to prepare fresh solutions.

A PANalytical X’Pert-PRO X-ray diffractometer (Almelo, The Netherlands) applying a Cu/Kα radiation (λ:1.54 Å) was used for X-ray-diffraction (XRD) analysis, and a Bruker Tensor II spectrometer (Bruker, Karlsruhe, Germany) was employed to capture the Fourier transform infrared (FT-IR) spectra. A MIRA3 scanning electron microscope coupled with an energy-dispersive X-ray spectroscopy (EDS) detector (Tescan, Brno, Czech Republic) was utilized for field-emission scanning electron microscopy (FE-SEM) (Tescan, Brno, Czech Republic) images and elemental analysis.

### 2.2. Solvents and Chemicals

All solvents and chemicals applied in our protocol were of analytical grade obtained from Merck and Sigma-Aldrich (Darmstadt, Germany). The phosphate buffer solution (PBS) was prepared with phosphoric acid and adjusted with NaOH to the desired pH value.

### 2.3. Preparation of Graphene-Co_3_O_4_ Nanocomposite

First, graphene oxide (GO) (20 mg) was dissolved in ethanol (20 mL) under 30-min ultra-sonication. Then, Co(NO_3_)_2_.6H_2_O (0.001 mol) was dissolved in ethanol (20 mL) while stirring for 30 min at ambient temperature. Next, the two prepared solutions were blended, 3.6 mL of an ammonia (NH_3_.H_2_O (wt. 25%)) solution was added dropwise, and the solution was subsequently transferred into a Teflon-lined stainless-steel autoclave and maintained at 180 °C for 24 h. After completion of the reaction, centrifugation was performed to collect the product, followed by rinsing with ethanol/deionized water. At last, the graphene-Co_3_O_4_ nanocomposite was oven-dried at 70 °C overnight.

### 2.4. Preparation of the Graphene-Co_3_O_4_/SPGE Sensor

A drop-casting technique was followed to fabricate the graphene-Co_3_O_4_/SPGE. Thus, a certain amount of as-prepared graphene-Co_3_O_4_ nanocomposite (1 mg) was dispersed in deionized water (1 mL) under 20-min ultra-sonication. Then, the well-dispersed suspension (4 µL) was coated on the SPGE surface dropwise and dried at the laboratory’s temperature.

The electrochemically active surface area (EASA) of the unmodified SPGE and graphene-Co_3_O_4_/SPGE was calculated from the cyclic voltammetry (CV) measurements in a 0.1 M KCl solution containing 1.0 mM K_3_[Fe(CN)_6_] as a redox probe at different scan rates. The value of the EASA was calculated according to the Randles-Sevcik equation [55]. The values of the EASA for the unmodified SPGE and graphene-Co_3_O_4_/SPGE are calculated as 0.05 and 0.16 cm^2^, respectively. The results reveal that the graphene-Co_3_O_4_/SPGE has a larger EASA than the unmodified SPGE.

### 2.5. Preparation of Real Samples

Urine samples were obtained from healthy volunteers and were stored in a refrigerator immediately after collection. Ten milliliters of each sample was centrifuged for 15 min at 2000 rpm. The supernatant was filtered out using a 0.45 μm filter. Then, varying volumes of the solution were transferred into 25 mL volumetric flasks and diluted to the mark with PBS (pH 7.0). The diluted urine sample was spiked with different amounts of morphine and diclofenac. The morphine and diclofenac contents were analyzed by the proposed method using the standard addition method.

One milliliter of morphine ampoule (labeled 10 mg per mL, Alborz Darou Company, Alborz Industrial City, Iran) was diluted to 10 mL with 0.1 M PBS (pH 7.0); then, varying volumes of the diluted solution were transferred into each of a series of 25 mL volumetric flasks and diluted to the mark with PBS. The analysis of morphine and diclofenac was performed using the standard addition method.

Five diclofenac tablets (labeled 50 mg per tablet, Darou Pakhsh Company, Tehran, Iran) were ground. Then, a tablet solution was prepared by dissolving 150 mg of the powder in 25 mL water by ultrasonication. Then, a different volume of the diluted solution was transferred into a 25 mL volumetric flask and diluted to the mark with PBS (pH 7.0). The morphine and diclofenac contents were analyzed by the proposed method using the standard addition method.

## 3. Results and Discussion

### 3.1. Characterization of Graphene-Co_3_O_4_ Nanocomposite

**Figure 1:**
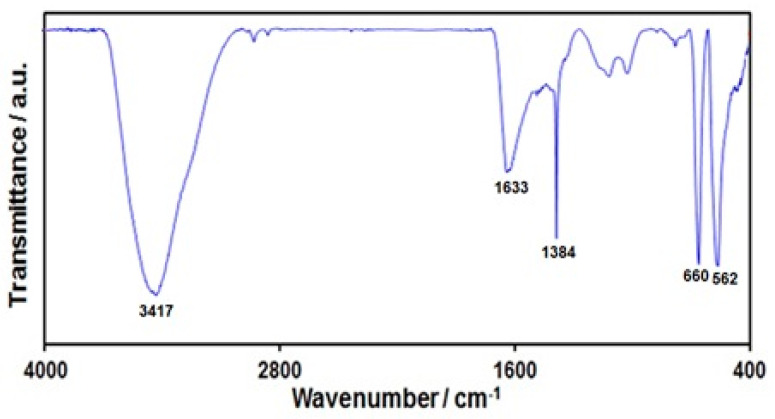
FT-IR spectrum of graphene-Co_3_O_4_ nanocomposite.

FT-IR analysis was performed to confirm the formation of graphene-Co_3_O_4_ nanocomposite (Figure 1). The wide absorption peak at 3417 cm^−1^ was representative of functional groups of OH. The peak at 1633 cm^−1^ related to aromatic C=C graphitic carbon domain vibration. The band at 1384 cm^−1^ related to OH deformation and bending vibration of interlayer water molecules [56]. The peaks at 562 and 660 cm^−1^ related to Co-O metal-oxygen bond stretching vibrations, approving Co_3_O_4_ nanoparticle formation.

**Figure 2:**
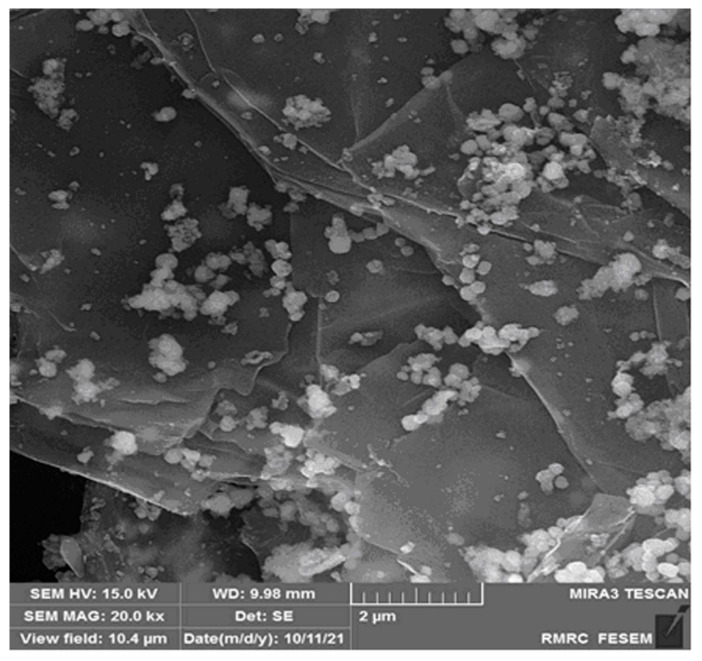
The FE-SEM image of graphene-Co_3_O_4_ nanocomposite.

The FE-SEM image was captured to explore the morphology and structure of the graphene-Co_3_O_4_ nanocomposite. Figure 2 shows the FE-SEM image of the graphene-Co_3_O_4_ nanocomposite. It can be seen that Co_3_O_4_ nanoparticles are anchored and densely dispersed on the surface of graphene nanosheets.

**Figure 3:**
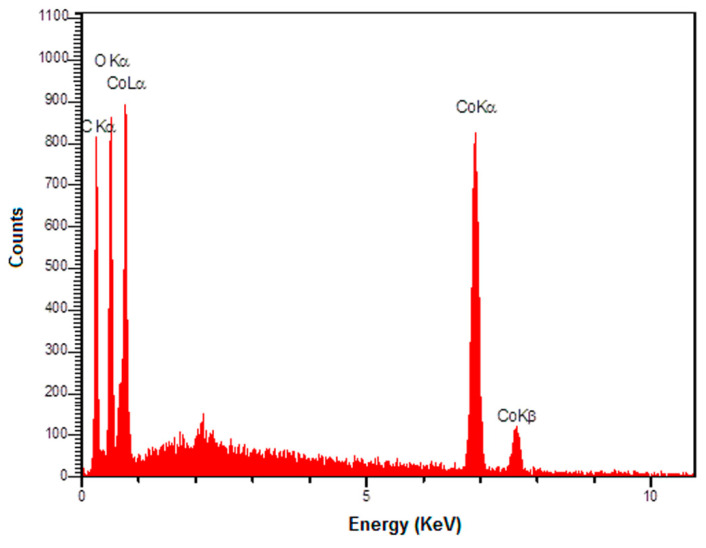
The EDS spectrum of graphene-Co_3_O_4_ nanocomposite.

The EDS spectrum (Figure 3) also shows the existence of C, O, and Co elements in the graphene-Co_3_O_4_ nanocomposite.

**Figure 4:**
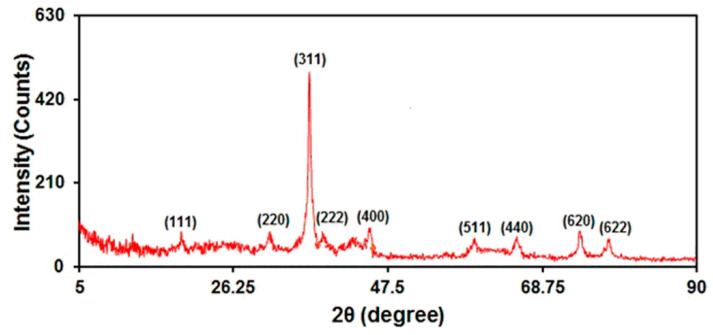
XRD pattern of graphene-Co_3_O_4_ nanocomposite.

The crystalline phase of the as-prepared graphene-Co_3_O_4_ nanocomposite was confirmed by XRD measurements (Figure 4). The obtained XRD patterns can be indexed according to the (111), (220), (311), (222), (400), (511), (440), (620), and (622) reflections of Co_3_O_4_ [JCPDS: 01-076-1802]. No distinct diffraction peak was observed for graphene, as the refraction of graphene is weaker than well-crystalline Co_3_O_4_.

### 3.2. Electrochemical Response of Morphine on Diverse Electrodes

**Figure 5:**
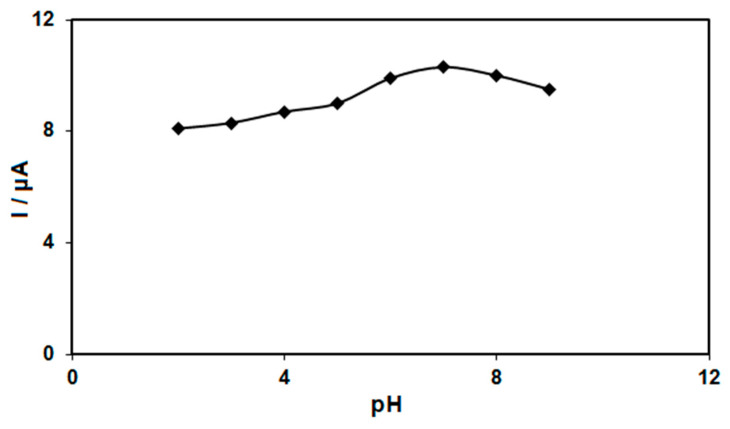
Plot of Ip vs. pH obtained from DPVs of graphene-Co_3_O_4_/SPGE in a solution containing 200.0 μM of morphine in 0.1 PBS with different pHs (2.0, 3.0, 4.0, 5.0, 6.0, 7.0, 8.0 and 9.0).

The electrochemical response of morphine oxidation in the 0.1 M PBS adjusted to variable pH values (2.0–9.0) was explored to determine the influence of the electrolyte solution’s pH. The results showed that the peak current of morphine oxidation depended on the pH value, so that it increased as the pH increased, reaching its maximum at a pH of 7.0, and then decreased with further increasing pH values (Figure 5). Hence, the pH value of 7.0 was considered to be the optimum for subsequent electrochemical determinations.

**Figure 6:**
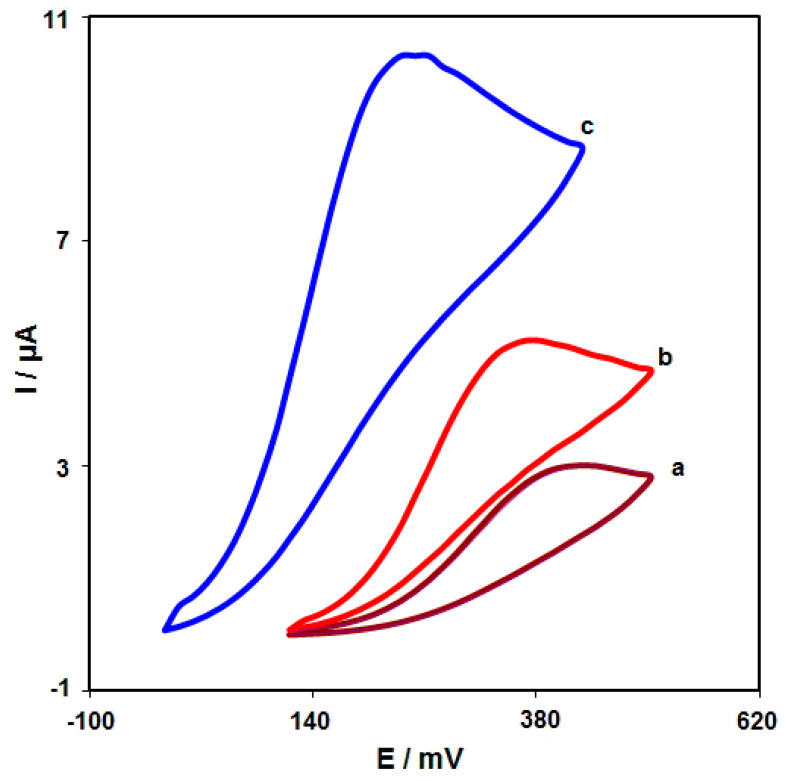
Cyclic voltammograms captured for oxidation of morphine (200.0 μM) in PBS (0.1 M; pH = 7.0) on: (a) unmodified SPGE; (b) graphene/SPGE; (c) graphene-Co_3_O_4_/SPGE with the scan rate of 50 mV/s.

CV was performed to clarify the electrochemical behavior of morphine on: (a) an unmodified SPGE; (b) a graphene/SPGE; (c) graphene-Co_3_O_4_/SPGE surfaces. Figure 6 compares the bare SPGE and the graphene-Co_3_O_4_/SPGE for 200.0 μM morphine oxidation in 0.1 M PBS at the pH value of 7.0. The morphine oxidation displayed a tiny and wide peak (3.0 μA) at the potential of 440 mV on the bare SPGE surface. Furthermore, the graphene/SPGE exhibited a shift in the peak current towards the more negative potentials (370 mV) by raising the amount of current (5.2 μA). The graphene-Co_3_O_4_-modified SPGE exhibited a shift in the peak current towards the more negative potentials (250 mV) by raising the amount of current (10.3 μA). Such an improvement can appear because of an appreciable catalytic impact of the graphene-Co_3_O_4_ nanocomposite for the morphine oxidation.

### 3.3. Effect of Scan Rate

**Figure 7:**
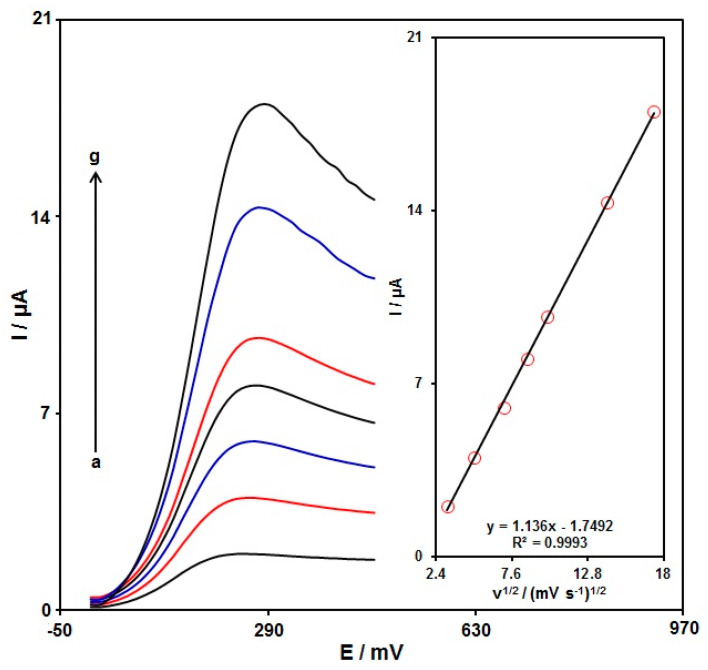
LSVs captured for the oxidation of morphine (100.0 μM) on the graphene-Co_3_O_4_/SPGE under variable scan rates (a–g: 10, 25, 50, 75, 100, 200, and 300 mV/s); Inset: the correlation of Ipa with ∪^1/2^.

The linear sweep voltammograms (LSVs) were recorded for the oxidation of morphine (100.0 μM) on the graphene-Co_3_O_4_/SPGE under variable scan rates (Figure 7). There was an apparent gradual elevation in the oxidation peak by raising scan rate ranging from 10 to 300 mV/s. As seen in Figure 7 (Inset), the anodic peak current (Ipa) had a linear association with the scan rate square root (∪^1/2^). The regression equation was obtained to be Ipa (µA) = 1.136 ∪^1/2^ (mV s^−1^)^1/2^ − 1.7492 (R^2^ = 0.9993), representing a controlled diffusion process of the morphine oxidation on the graphene-Co_3_O_4_/SPGE.

### 3.4. Chronoamperometric Analysis

**Figure 8:**
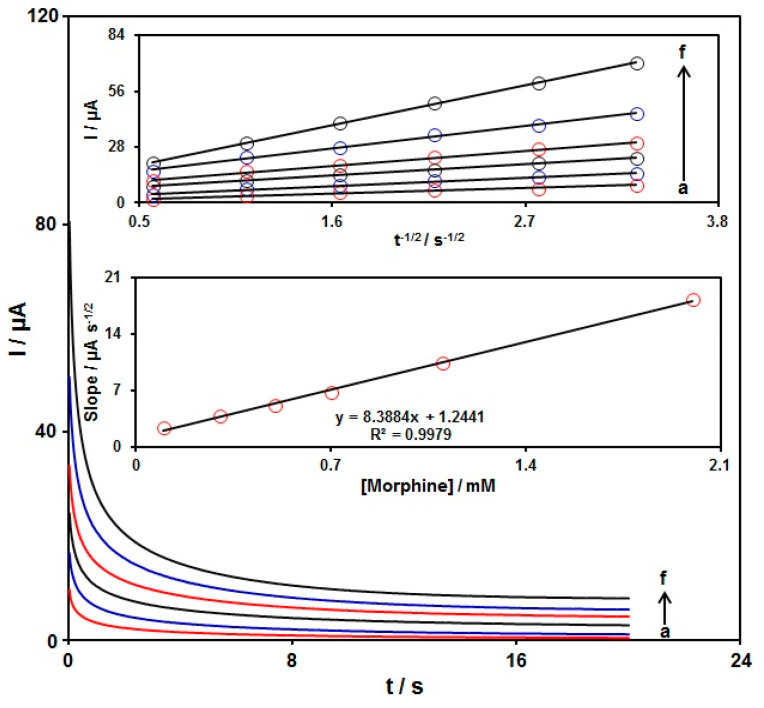
Chronoamperometric behavior of graphene-Co_3_O_4_/SPGE in PBS (0.1 M; pH = 7.00) at a potential of 300 mV for variable morphine contents (a–f: 0.1, 0.3, 0.5, 0.7, 1.1, and 2.0 mM); Insets: (top) plots of I vs. t^−1/2^; (bottom) plots of the slopes from the straight lines vs. morphine level.

Chronoamperometry was used to explore the morphine catalytic oxidation on the graphene-Co_3_O_4_/SPGE surface. Chronoamperometric analysis was performed for variable morphine contents on the graphene-Co_3_O_4_/SPGE at the working electrode potential of 300 mV. The chronoamperograms captured for variable morphine contents on the graphene-Co_3_O_4_/SPGE are seen in Figure 8. The Cottrell equation explains the current (I) for electrochemical reaction of an electroactive material with a D value (diffusion coefficient) under a mass transport limited condition. Figure 8 shows a linear relationship of the I value with t^−1/2^ for the oxidation of variable morphine contents. The slopes from the obtained straight lines were plotted against variable morphine contents (Figure 8). The plotted slope and Cottrell equation estimated the D value to be 6.0 × 10^−6^ cm^2^/s for morphine.

### 3.5. DPV Analysis of Morphine

**Figure 9:**
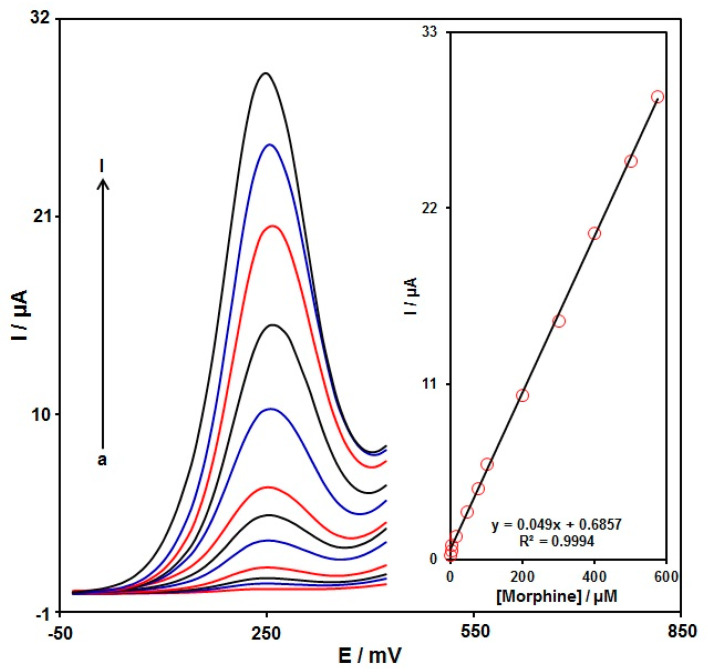
DPVs captured for the oxidation of variable morphine contents on the graphene-Co_3_O_4_/SPGE under variable contents (a–l: 0.02, 0.25, 2.5, 15.0, 45.0, 75.0, 100.0, 200.0, 300.0, 400.0, 500.0, and 575.0 μM); Inset: Calibration curve of voltammetric response (Ipa) against morphine level.

DPV analysis was performed for variable morphine contents to explore the linear dynamic range, limit of detection (LOD), and sensitivity of the graphene-Co_3_O_4_/SPGE under optimized experimental circumstances (Figure 9) (step potential = 0.01 V and pulse amplitude = 0.025 V). As expected, the elevation in the morphine level enhanced the peak current. Figure 9 (Inset) shows a linear proportion of the oxidation peak currents to variable morphine contents (0.02 μM to 575.0 μM) with the linear regression equation of Ipa (μA) = 0.049 C-morphine + 0.6857 (R^2^ = 0.9994), and the sensitivity of 0.4 µA/(µM cm^2^). In the equation of LOD = 3 σ/m, the σ stands for the standard deviation of the response for blank solution, and the m for the slope from the standard graph. The LOD was estimated at 0.007 μM for morphine determination on the graphene-Co_3_O_4_/SPGE. Table 1 compares the efficiency of the morphine sensor prepared by the graphene-Co_3_O_4_-nanocomposite-modified SPGE and other reported works.

### 3.6. DPV Analysis for Determination of Morphine in the Presence of Diclofenac

**Figure 10:**
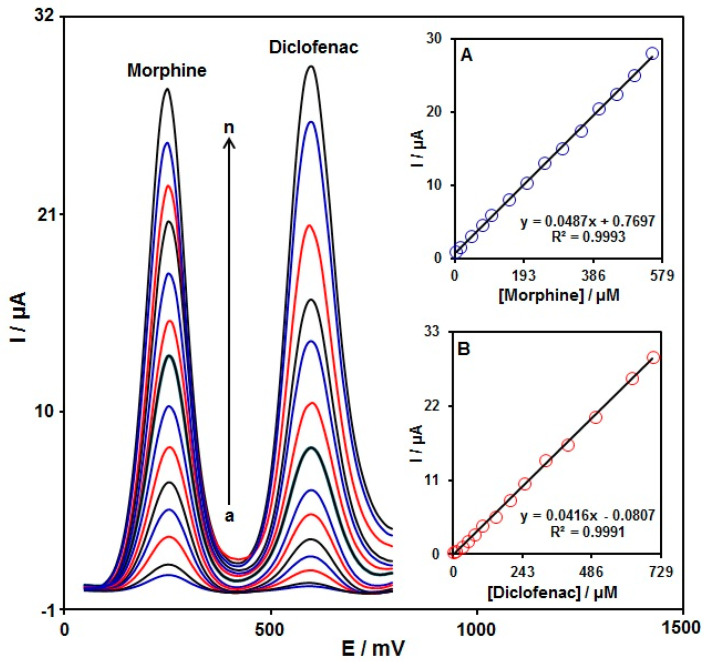
DPVs of graphene-Co_3_O_4_/SPGE in PBS (0.1 M, pH = 7.0) with variable morphine concentrations (a–n: 2.5, 15.0, 45.0, 75.0, 100.0, 150.0, 200.0, 250.0, 300.0, 350.0, 400.0, 450.0, 500.0, and 550.0 μM) and diclofenac (a–n: 2.5, 10.0, 30.0, 50.0, 75.0, 100.0, 150.0, 200.0, 250.0, 325.0, 400.0, 500.0, 625.0, and 700.0 μM). Inserts: (**A**) plot of peak current versus morphine concentration; (**B**) plot of peak current versus diclofenac concentration.

To confirm the ability of the graphene-Co_3_O_4_/SPGE for co-detection of morphine with diclofenac, the electrochemical responses of these analytes were detected by simultaneously changing the concentration of both analytes in PBS at a pH of 7.0. As seen in Figure 10, with the concurrent change in their concentrations, two non-interference peaks were found on DPV curves (step potential = 0.01 V and pulse amplitude = 0.025 V). The peak currents of both morphine and diclofenac oxidation displayed a linear elevation with the respective concentrations (morphine concentration range between 2.5 μM and 550.0 μM and diclofenac concentration range between 2.5 μM and 700.0 μM) (Figure 10A,B). The intensity of the peak current showed good linearity with the target concentration change, meaning the possibility of detecting morphine and diclofenac in the blended solution.

### 3.7. Reproducibility, and Stability

To test the reproducibility, seven graphene-Co_3_O_4_/SPGEs produced by the same procedures were applied to measure 50.0 µM morphine under identical circumstances; the obtained relative standard deviation (RSD) of 6.1% suggested commendable reproducibility.

To test the graphene-Co_3_O_4_/SPGE’s stability, the current responses of 50.0 μM morphine were measured following 20-day storage of the sensor at ambient temperature. The decrease in the peak current of morphine to 97.1% of its original response demonstrated appreciable stability.

### 3.8. Interference Study

The selectivity studies of the graphene-Co_3_O_4_/SPGE for the determination of 50.0 μM morphine were performed in the presence of different interfering substances. The tolerance limit was defined as the maximum concentration of the interfering substance that caused an approximately ±5% relative error in the determination. The results revealed that 480-fold of Na^+^, Mg^2+^, Cl^−^, Br^−^, NH_4_^+^, fructose, glucose, and lactose; 300-fold of histidine, phenyl alanine, alanine, methionine, glycine, methanol, ethanol, and tryptophan; 230-fold of ascorbic acid (after removal with ascorbic oxidase), thiourea, tyrosine, cysteine, and acetaminophen; and 10-fold of dopamine, uric acid, and methyldopa did not show interference in determination. Those results confirm the suitable selectivity of the proposed sensor for the determination of morphine.

### 3.9. Analysis of Real Specimens

The practical applicability of the graphene-Co_3_O_4_/SPGE was tested by sensing morphine and diclofenac in morphine ampoule, diclofenac tablet, and urine specimens using the DPV procedure and the standard addition method, the results of which can be seen in Table 2. The recovery rate was between 96.5% and 104.0%, and all RSD values were ≤3.5%. According to the experimental results, the graphene-Co_3_O_4_/SPGE sensor possessed a high potential for practical applicability.

## 4. Conclusions

The present work examined a simple and sensitive electrochemical morphine sensor using a graphene-Co_3_O_4_-nanocomposite-modified SPGE. The graphene-Co_3_O_4_/SPGE sensor showed excellent activity towards oxidation of morphine at a low overpotential ∼250 mV with a high current response of around 10.3 μA. The graphene-Co_3_O_4_/SPGE had a wider linear response in concentration ranging from 0.02 to 575.0 μM with an LOD as narrow as 0.007 μM. Furthermore, by separating the peaks of morphine and diclofenac oxidation with a potential difference of 350 mV, it was possible to measure morphine in the combination with diclofenac using the modified sensor. Finally, the proposed electrode was successfully employed for the determination of diclofenac and morphine in real specimens such as diclofenac tablet, morphine ampoule, and urine applying standard addition method with acceptable recoveries. The novelty of this work concerns the observed synergistic effect of graphene nanosheets and a Co_3_O_4_ nanoparticles-modified electrode for enhanced electrochemical sensing of morphine in the presence of diclofenac.

## Figures and Tables

**Table 1:** Comparison of the efficiency of the graphene-Co3O4/SPGE sensor with other reported modified electrodes for morphine determination.

Electrochemical Sensor	Electrochemical Method	Linear Range	LOD	Ref.
ZnO-multi-walled carbon nanotubes (MWCNTs)-ionic liquid/carbon-paste electrode (CPE)	-	0.1–700.0 μM	0.06 μM	[1]
Polydopamine-modified MWCNTs-glassy Carbon Electrode (GCE)	DPV	0.075–75.0 μM	0.06 μM	[37]
Poly(cetyltrimethylammonium bromide)/graphene oxide/GCE	DPV	50–60 μM	0.36 μM	[38]
Au Nanoparticles/CPE	DPV	4.0 × 10^−7^–2.0 × 10^−4^ M	4.21 nM	[39]
Au nanoparticles and Co phthalocyanine/CPE	DPV	4.0 × 10^−7^–9.0 × 10^−4^ M	5.48 × 10^−9^ M	[40]
Ordered mesoporous carbon/GCE	CV	0.1–20 μM	10 nM	[44]
Graphene-Co3O4/SPGE	DPV	0.02–575.0 μM	0.007 μM	This work

**Table 2:** Voltammetric sensing of morphine and diclofenac in real specimens using graphene-Co_3_O_4_/SPGE. All concentrations are in µA (n = 5).

Sample	Spiked (μM)		Found (μM)		Recovery (%)		R.S.D. (%)	
	Morphine	Diclofenac	Morphine	Diclofenac	Morphine	Diclofenac	Morphine	Diclofenac
Morphine ampoule	0	0	3.0	-	-	-	3.2	-
	1.0	5.0	3.9	5.1	97.5	102.0	2.7	3.5
	2.0	7.0	5.1	6.9	102.0	98.6	1.9	2.1
	3.0	9.0	6.2	8.8	103.3	97.8	2.4	2.6
	4.0	11.0	6.9	11.1	98.6	100.9	3.0	1.8
Diclofenac tablet	0	0	-	4.0	-	-	-	2.9
	5.0	1.0	4.9	5.1	98.0	102.0	3.0	2.4
	7.5	3.0	7.6	6.8	101.3	97.1	2.0	3.3
	10.0	5.0	9.9	9.1	99.0	101.1	2.9	1.7
	12.5	7.0	13.0	10.9	104.0	99.0	2.3	2.3
Urine	0	0	-	-	-	-	-	-
	4.0	5.5	3.9	5.6	97.5	101.8	3.1	1.8
	6.0	7.5	6.2	7.4	103.3	98.7	2.8	2.9
	8.0	9.5	8.1	9.8	101.25	103.2	1.9	3.5
	10.0	11.5	9.9	11.1	99.0	96.5	2.2	2.4

## Data Availability

The data presented in this study are available upon request from the corresponding authors.
